# Platypnea-orthodeoxia syndrome: echocardiography-guided percutaneous treatment

**DOI:** 10.31744/einstein_journal/2025RC1568

**Published:** 2025-10-03

**Authors:** João de Azevedo, Fernando Hideki Sakamoto, Sofia Alves Figueiredo Faustino, César Henrique Moraes, Ítalo Menezes Ferreira, Ana Clara Tude Rodrigues, Claudio Henrique Fischer, Samira Saad Morhy, Adriana Venturini Reche, Adriano Caixeta, Marcelo Luiz Campos Vieira

**Affiliations:** 1 Hospital Israelita Albert Einstein São Paulo SP Brazil Hospital Israelita Albert Einstein, São Paulo, SP, Brazil.

**Keywords:** Platypnea orthodeoxia syndrome, Orthodeoxia, Foramen ovale, patent, Dyspnea, Oxygen saturation, Echocardiography

## Abstract

Platypnea-orthodeoxia syndrome, first described in 1949, is a rare clinical condition characterized by positional dyspnea and oxygen desaturation in the upright position that improves when lying down. Herein, we report the case of an 86-year-old patient who experienced episodes of dyspnea on assuming an upright position, which limited her daily activities. The patient's oxygen saturation was 82% in the standing position and 91% in the supine position. During diagnostic investigation, a patent foramen ovale was identified using transesophageal echocardiography. The patient underwent percutaneous patent foramen ovale closure with an Amplatzer device, which resulted in significant improvement in her symptoms and oxygen saturation. Diagnosis of this condition requires a high index of clinical suspicion and is confirmed when the syndrome's signs and symptoms correlate with the presence of a right-to-left interatrial shunt. It is predominantly diagnosed using echocardiography with agitated saline injection.

## INTRODUCTION

Platypnea-orthodeoxia syndrome (POS) is a rare and frequently underdiagnosed clinical entity defined by the occurrence of dyspnea and arterial oxygen desaturation in the upright position, which improves in the supine position.^(1)^ Although a patent foramen ovale (PFO) is a common congenital anomaly, present in approximately 25-30% of the population,^(2)^ it rarely causes symptoms because the left atrial pressure is physiologically higher than the right atrial pressure. However, certain anatomical or functional changes can facilitate a right-to-left shunt, triggering the hypoxemia characteristic of this syndrome. The diagnosis of POS requires a high degree of clinical suspicion and an accurate investigation to confirm the presence and relevance of an interatrial shunt, with transesophageal echocardiography being the gold standard method.^(3)^ This report presents a case of POS secondary to PFO, highlighting the diagnostic process and the efficacy of percutaneous therapy.

## CASE REPORT

An 86-year-old female patient with dyslipidemia presented with dizziness and dyspnea upon assuming an upright position, which led to limitations of her daily activities and a preference for the supine position. Her oxygen saturation was 82% in the standing position and 91% in the supine position, prompting hospitalization for desaturation and a targeted investigation for POS.

**Figure 1:**
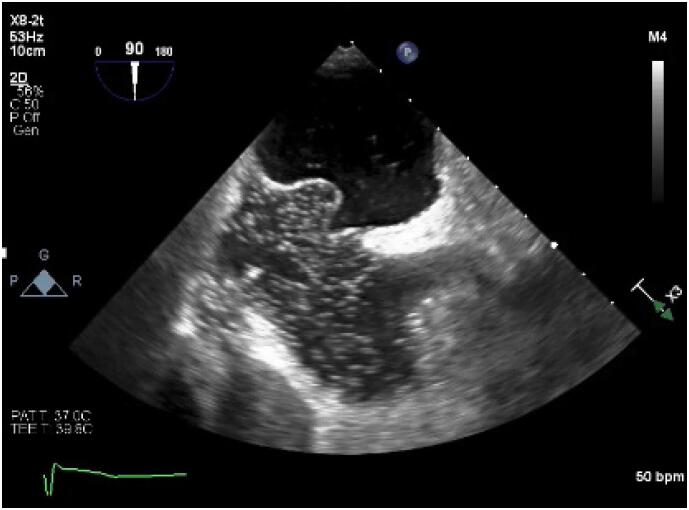
Transesophageal echocardiogram showing an aneurysmal interatrial septum with bubble passage from the right atrium to the left atrium

Laboratory tests and electrocardiography revealed no significant abnormalities. Chest computed tomography angiography revealed normal pulmonary parenchyma without signs of infection or inflammation, right hemidiaphragm elevation, and no pulmonary embolism. Transesophageal echocardiography (TEE) identified an aneurysmal fossa ovalis membrane with right-to-left interatrial flow. An agitated saline injection demonstrated significant bubble passage, confirming a PFO (Figure 1).

Cranial magnetic resonance imaging revealed ischemic sequelae in the left cerebellar hemisphere. Additionally, transcranial Doppler revealed numerous gaseous emboli in the cerebral circulation, especially after the Valsalva maneuver, demonstrating a curtain sign (maximum cerebral embolization) and high-conductance right-to-left communication.

**Figure 2:**
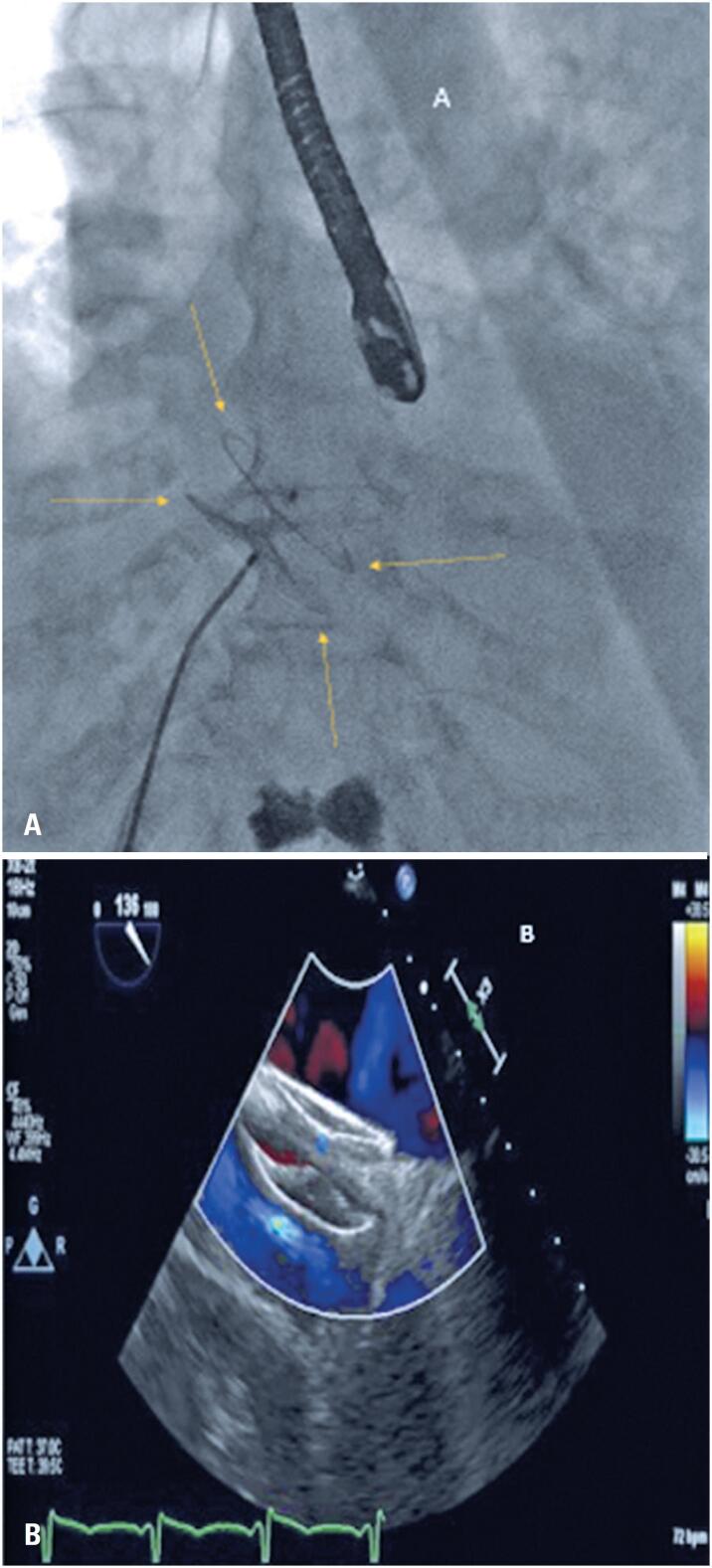
A) Fluoroscopy showing a 40-mm Amplatzer PFO device; B) Transesophageal echocardiogram with color Doppler showing minimal residual shunting after the device was deployed

The patient was referred for percutaneous PFO closure guided by TEE. During the procedure, blood samples from the pulmonary veins and left atrium were collected, confirming significant shunting with a maximum PaO_2_ of 144mmHg and 99% hemoglobin saturation in the pulmonary vein and 113mmHg and 97% in the left atrium. A 40mm Amplatzer PFO device was successfully deployed (Figure 2) with minimal residual shunting around the device. After the procedure, the patient's platypnea symptoms improved, with her oxygen saturation increasing to 93% while standing. The patient was discharged in good condition.

## DISCUSSION

Platypnea-orthodeoxia syndrome was first described in 1949 in a patient with a post-traumatic intrathoracic arteriovenous shunt.^(4)^ It is a rare clinical entity characterized by positional dyspnea (platypnea) and oxygen desaturation (orthodeoxia) in the upright position, which improves in the supine position. It is defined as a decrease in arterial oxygen pressure (PaO_2_) of at least 4mmHg or in oxygen saturation (SaO_2_) of at least 5% from the supine to the upright position.^(1)^

The etiology of POS includes several conditions subdivided by shunt topography and hypoxemia mechanisms and is classified as intracardiac or extracardiac.^(5)^ Intracardiac shunts, particularly right-to-left interatrial shunting via a PFO or atrial septal defects, are the most common etiologies. These defects allows deoxygenated venous blood to mix with oxygenated arterial blood, reducing systemic oxygenation and resulting in dyspnea.^(6)^

A PFO results from incomplete fusion of the septum secundum and septum primum during embryogenesis^(2)^ and occurs in 25-30% of the population, typically without symptoms. Isolated PFO usually does not cause right-to-left shunting due to higher left atrial pressure (5-8mmHg) relative to the right atrium. However, conditions that increase right atrial pressure may overcome this functional closure, allowing shunting. Additional mechanisms that enable shunting despite normal right atrial pressure include prominent Eustachian valves, aortic aneurysms, diaphragmatic paralysis, paresophageal hernias, and upright positioning. In the present case, TEE with agitated saline confirmed that PFO was the underlying cause of POS.^(3,7)^

## CONCLUSION

The diagnosis of platypnea-orthodeoxia syndrome requires a high index of suspicion. As it is underrecognized, platypnea-orthodeoxia syndrome is often underdiagnosed. Evaluation should begin with clinical history, physical examination, and oxygen saturation measurements in both supine and upright positions. Diagnostic approaches depend on etiology, with patent foramen ovale being the most commonly reported cause. Transesophageal echocardiography with agitated saline remains the gold standard for the accurate diagnosis of patent foramen ovale.

## References

[1] Agrawal A, Palkar A, Talwar A (2017). The multiple dimensions of platypneaorthodeoxia syndrome: a review. Respir Med..

[2] Meier B, Lock JE (2003). Contemporary management of patent foramen ovale. Circulation.

[3] Bertaux G, Eicher JC, Petit A, D’Allounes C, Wolf JE, Granier I (1992). Diagnosis of patent foramen ovale by contrast transesophageal echocardiography. J Am Coll Cardiol.

[4] Cheng TO (1999). Platypnea-Orthodeoxia Syndrome: Etiology, Differential Diagnosis, and Management. Catheter Cardiovasc Interv.

[5] Blanc P, Auriol A, Lacassagne L, Cérénys M, Le Bidois J, Rousseau H (2010). Diagnosis of platypnea-orthodeoxia syndrome using dynamic CT: a case report. J Radiol Case Rep.

[6] Thakkar AN, Mautner RD, Park DS, Mautner SL (2014). Atrial septal defect presenting as platypnea-orthodeoxia syndrome. Circulation.

[7] Konstantinides S, Geibel A, Olschewski M, Görnandt L, Roskamm H, Katus HA (1998). Atrioventricular and intrapulmonary shunts in patients with hepatic cirrhosis: prevalence and clinical relevance. Hepatology.

